# Supplementary figures and images for: Translation control by altered start codon usage as a means of modulating the general stress response and virulence in Listeria monocytogenes
Source: PLoS Genet. 2026 Apr 6;22(4):e1011851. doi: 10.1371/journal.pgen.1011851 (PMC13068326; doi:10.1371/journal.pgen.1011851)

S1 Fig


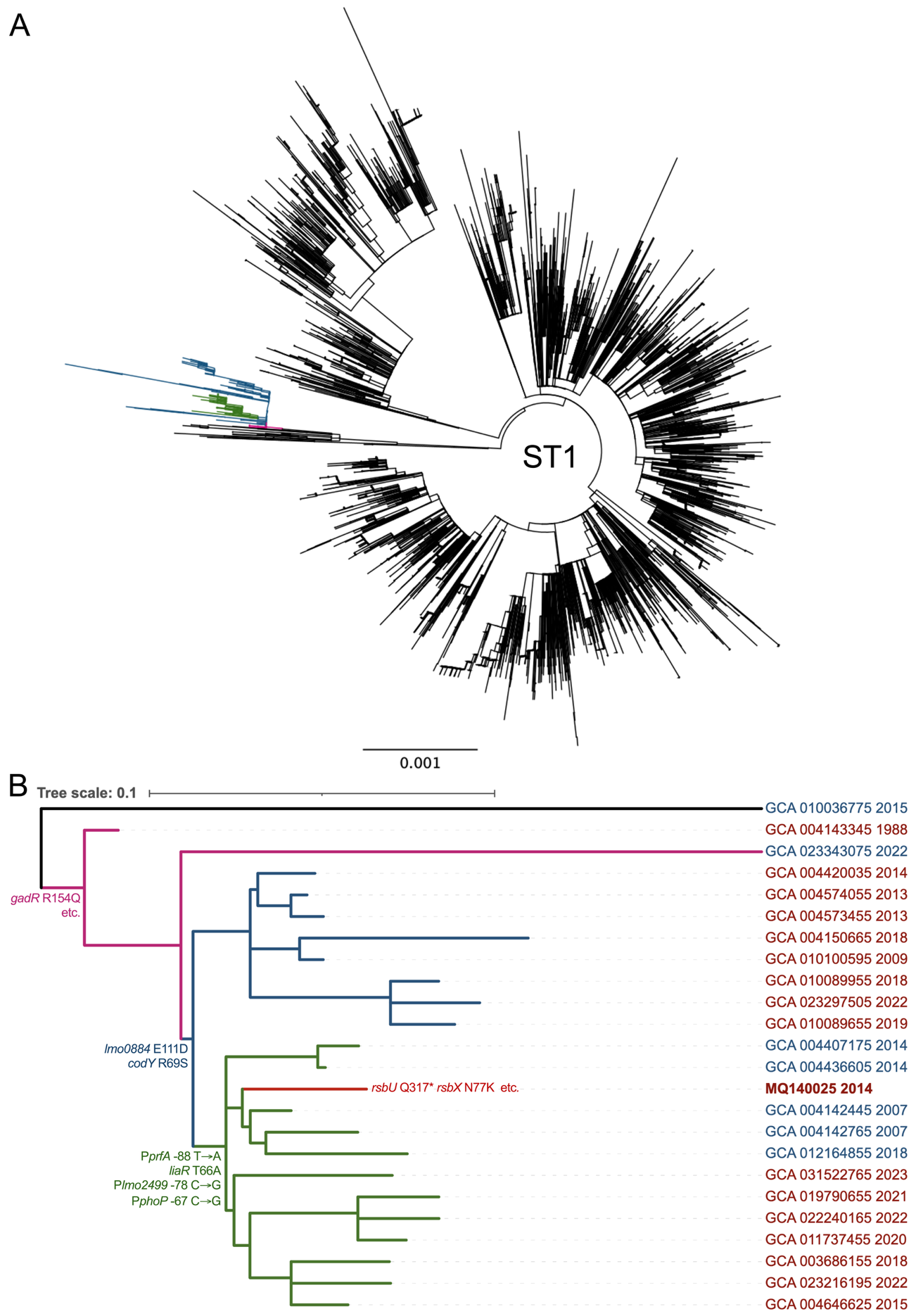

Supplement: S1 Fig — (A) Core-genome based phylogeny of ST1 5446 genomes. Genetic clade in which MQ140025 was placed is highlighted in colour. (B) Detailed illustration of MQ140025 and its close relatives within coloured branch in panel A. (DOCX) [file pgen.1011851.s002.docx]

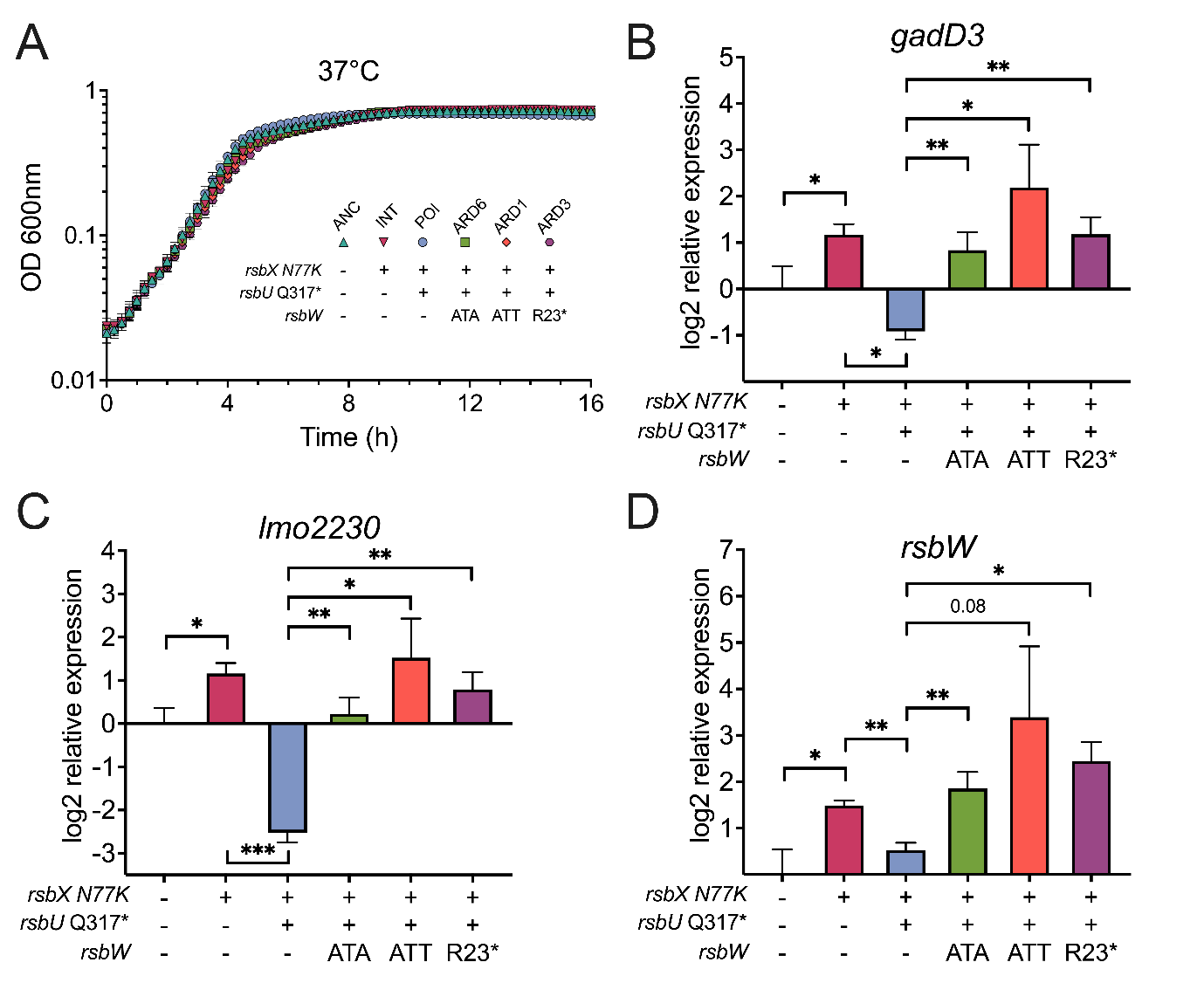
S2 Fig

Supplement: S2 Fig — Growth curves at 37°C are shown (A). Transcripts levels of gadD3 (B), lmo2230 (C) and rsbW (D) at stationary phase were measured and expressed relative to MQ140025 strain with wild type sigB operon using 16S as reference gene. Transcripts levels of sigB at stationary phase were measured and expressed relative to MQ140025 strain with wild type sigB operon using rsbW as reference gene (E). For transcriptional analysis, three independent experiments were carried out and each with technical duplicates. All statistical significance was calculated between each strain to MQ140025 using paired two-tailed t-test (ns, not significant; *, P < 0.05; **, P < 0.01; and ***, P < 0.001). (DOCX) [file pgen.1011851.s003.docx]
