# Supplementary material for: Translation control by altered start codon usage as a means of modulating the general stress response and virulence in Listeria monocytogenes
Source: PLoS Genet. 2026 Apr 6;22(4):e1011851. doi: 10.1371/journal.pgen.1011851 (PMC13068326; doi:10.1371/journal.pgen.1011851)
Supplement: S1 Data — (ZIP) [file pgen.1011851.s005.zip › Wu_2026_PG_fulldata/Fig5C.docx]

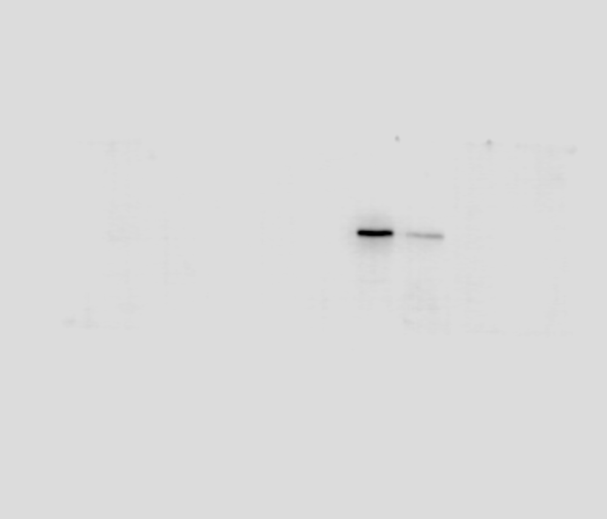
Independent experiment (biological repeat) 1


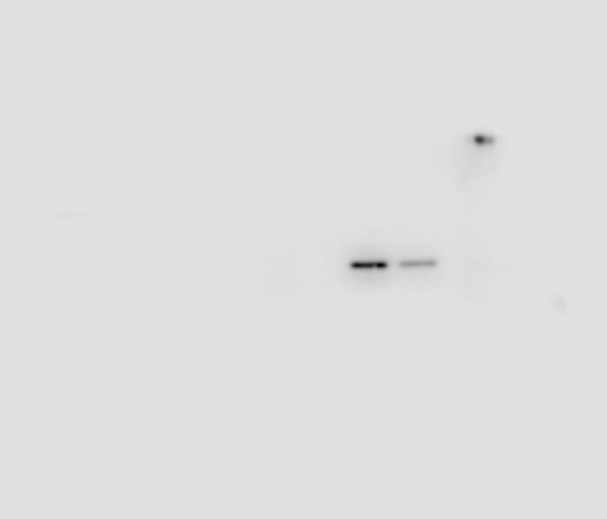
Independent experiment (biological repeat) 2


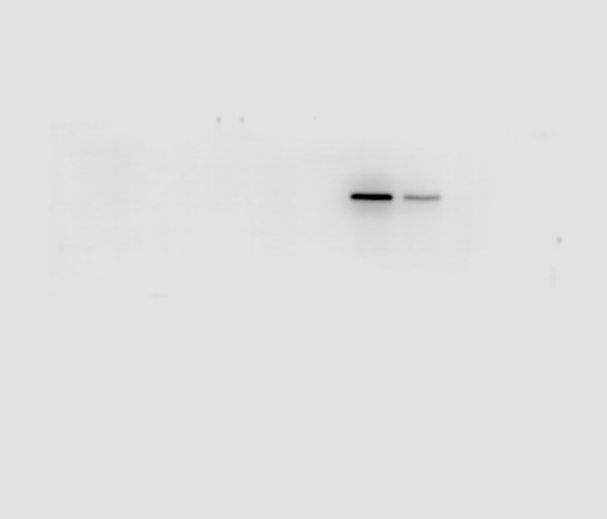
Independent experiment (biological repeat) 3
