# Supplementary material for: Translation control by altered start codon usage as a means of modulating the general stress response and virulence in Listeria monocytogenes
Source: PLoS Genet. 2026 Apr 6;22(4):e1011851. doi: 10.1371/journal.pgen.1011851 (PMC13068326; doi:10.1371/journal.pgen.1011851)
Supplement: S2 Data — Start codon analysis output for all coding sequence analysed from a total of 60,690 L. monocytogenes genome sequences.Each file is tabular format output generated with tblastn, mlst, and start codon analysis.The columns are in such order:“tblastn subject sequence id”, “tblastn subject start”, “tblastn subject end”, “tblastn subjectlength”, “tblastn percentage iden:ty”, “tblastn query length”, “tblastn length”, “Genome filename”, “Start codon usage”. (PDF) [file pgen.1011851.s006.pdf]

Dear editor and reviewers:

In compliance with *Plos GENETICS* data availability policy, we disclose the relevant data for manuscript entitled: “Translation control by altered start codon usage as a means of modulating the general stress response and virulence in *Listeria monocytogenes*.” by Jialun Wu, Claire Kelly, Brenda Chanza, Ashley Reade, Catherine M. Burgess, and Conor O’Byrne.

The data had been stored at Zenodo database: https://doi.org/10.5281/zenodo.19196202

The nature of this data is a compressed folder contains the start codon analysis output for all coding sequence analysed from a total of 60,690 *L. monocytogenes* genome sequences. Each file is the tabular format output generated with tblastn, mlst, and start codon analysis. The columns are in such order:

“tblastn subject sequence id”, “tblastn subject start”, “tblastn subject end”, “tblastn subject length”, “tblastn percentage identity”, “tblastn query length” , “tblastn length”, “Genome file name”, “Start codon usage”

Best regards,

Jialun
