# Supplementary material for: Translation control by altered start codon usage as a means of modulating the general stress response and virulence in Listeria monocytogenes
Source: PLoS Genet. 2026 Apr 6;22(4):e1011851. doi: 10.1371/journal.pgen.1011851 (PMC13068326; doi:10.1371/journal.pgen.1011851)
Supplement: Attachment — Submitted filename: Point-by-point_response.pdf [file pgen.1011851.s008.pdf]

Editors' suggestions:
This reviewer mentioned several additional experiments that would strengthen and validate the findings. The one I would strongly consider is comment #4: "To validate their results, the authors need to reconstruct the *rsbW* start codon mutations in the parental background. Ideally, these mutations also should be transplanted into a SigB+ reference strain to determine their effect on SigB activation in a SigB+ background."
The authors thank editor for this excellent suggestion, and agree that this will help to strengthen the findings. The authors have explicitly addressed this concern as part of responses to reviewer 2. All responses by the authors are in red, with Ln numbers refer to clean version of resubmission.

Point-by-point response to reviewers:

Reviewers' comments:

Reviewer #1: In the research article titled "Translation control by altered start codon usage as a means of modulating the general stress response and virulence in Listeria monocytogenes.", the authors have employed an in vitro evolution experiment to genetically dissect the ability of the reference strain to adapt to enhanced acid stress. They concluded that the acid-resistant phenotypes of the reference strain variants were due to mutationally acquired start codon variations in the SigB regulon, specifically in the rsbW gene, which allowed for translational control of RsbW expression and thereby modulation of SigB activity. Additionally, the authors analyzed all publicly available genomes of Listeria monocytogenes strains to gauge better the differences in virulence and stress response gene expression levels resulting from flexible start codon usage.
Overall, the study is well-designed, and the hypothesis is well-tested. However, I had some comments and suggestions for the authors that would further improve the rigor of this study and the representation of the data:
The authors thank reviewer for carefully evaluating the manuscript and providing constructive comments and suggestions that help to improve the rigor and readability of this manuscript. The authors have now included additional data and modified the manuscript as recommended.
• Fig. 1A, B, and the text in lines 128-139 describing the results are unclear to me. Figure 1A shows that the cultures were subjected to daily cycles of stress at either pH 3 or pH 2.5 for 4 days. However, in Figure 1B, it seems that the pH 3 stress was given for the first two days, followed by pH 2.5 stress for days 3 and 4. Could the authors please provide more clarification as to which of the two approaches was used? Authors also need to mention in the results that the duration of acid stress was steadily increased on subsequent days for recoverable samples.
The authors thank reviewer 1 for pointing out this issue. We have revised this paragraph and clarified the change of acid inactivation conditions, and specified which sample was used to continue with the evolution from day 3 to day 4 (Ln134-148).
• Have the authors measured the transcript levels of sigB in the ARDs? It would be helpful to include this data as an additional panel in Fig. 2, which would further support the notion that the observed phenotypes resulted from increased activity rather than elevated levels of SigB.
The authors agree that measuring transcripts level of *sigB* (along with *rsbW*) would provide additional information that may help to understand the regulation of SigB activity in ARDs.
In *L. monocytogenes*, the transcription of *rsbW-sigB* is driven by a housekeeping promoter (SigA-dependent) upstream of *rsbR* (thus a *rsbRSTU-VW-sigB-rsbX* transcript) and a SigB-dependent promoter upsteam of *rsbV* (thus a *rsbVW--sigB-rsbX* transcript). Due to this partial positive auto-regulation of *sigB*, it is difficult to conclude the causations between upregulated *sigB* transcription and increased SigB translation. Furthermore, since SigB activity is regulated through a direct interaction with RsbW, higher SigB expression level does not necessarily correlate with higher SigB activity (reflected by upregulation of SigB-dependent genes). Therefore, we decided to measure transcript levels of *rsbW* and *sigB*, and determine their relative abundancies.

We have now included *rsbW* and *sigB* transcript measurements as part of additional Figure 4, using 16S and rsbW as reference genes, respectively. And the relevance of this data has been discussed at Ln274-276.

• In Fig. 4C, the – and + IPTG labels appear to be swapped. Please correct this. The authors also need to include a control showing that SigB levels were not affected -/+ IPTG. Alternatively, the authors may include a transcriptional reporter plot for sigB in the ARDs to illustrate this point.

The authors thank reviewer for pointing out this mistake, the label has been corrected now. These reporters were designed to test how the usage of different start codon (alone) influences translation efficiency. To avoid the influence from *sigB* operon autoregulation (as mentioned in answer to the previous question), we selected a plasmid (pIMK3) with SigB-independent promoter, and avoided the expression of any functional component that influences SigB activity. These reporters only express 15 aa C-terminal peptide from RsbV and an eGFP fused with 6 aa residues from N-terminal of RsbW upon IPTG induction. Additionally, these reporters were introduced to a SigB$^-$ strain at a locus that has no functional relevance to SigB activity or *sigB* operon. Therefore, the authors do not expect differential SigB activities among these conditions. Nevertheless, the authors realised that this wasn't made very clear and therefore, the design of these reporters has been further explained at Ln281-283.

• As part of the Fig. 4D illustration, it would be helpful to readers if the authors included more details of the SigB regulon (for example, the binding of SigB to the P2 promoter of prfA), as this provides context for subsequent analyses.

The authors thank the reviewer for this suggestion, indeed this will help readers to comprehend the role of SigB and provide context for subsequent analysis. This figure (now Figure 5) has been revised including *prfA* (P2) and several other members of SigB regulon that are important for stress response and virulence as examples.

• Information about the statistical analyses performed is missing in the figure legends of both Fig. 4 and 5. Please include these.

The authors thank the reviewer for pointing this out, the statistical analyses have been described in both figure legends now (Ln972-973, Ln981-983).

• Minor:

o Line 51: Remove "available".

This has been corrected, Ln54.

o Line 152: Typo "permanent"

This has been corrected, Ln159.

o Line 344: Typo "ancestral"

This has been corrected, Ln395.

o Line 831-832: three "parallel cultures"….were "repeatedly" exposed…

This has been corrected, Ln912.

Reviewer #2: The manuscript by Wu et al. describes an interesting observation regarding the occurrence of adaptive mutations in the regulatory circuit controlling activity of the alternative sigma factor SigB in the bacterium Listeria monocytogenes. SigB is a critical regulator of the virulence of this pathogen and mutations in the genes controlling its activity have recently been shown to explain differences between hypo- and hypervirulent sublineages (PMID: 39578578). Thus, the authors address a significant topic. The paper mainly describes suppressor mutants that arose when a clinical strain with lowered SigB activity (due to mutations in the rsbU and rsbX genes of the sigB operon) is challenged with acid stress, for which the SigB response is required to handle this. These suppressors were more resistant to acid stress, both at pH 3 and pH 2.5, and some of them formed smaller colonies on agar plates and showed low motility, resembling known small colony variants with increased SigB activity. To test the hypothesis that SigB activity is restored in these suppressors, the authors quantified transcription of known SigB-dependent genes. These genes (inlA, lmo2230) were in fact upregulated in most suppressors, whereas upregulation of SigB-independent acid stress response genes was not found to be upregulated (agu). Genome sequencing of the SigB-deficient clinical isolate and several acid resistant suppressors confirmed the rsbU *317Q mutation in the parental strain and identified

rsbW mutations in the acid resistant suppressors. The mutations found in rsbW were either nonsense mutations or mutations affecting the rsbW start codon (which at the same time affect the C-terminus of the upstream gene rsbV). To test the effect of the start codon mutations found on translation efficiency of rsbW, a reporter was constructed that contained the N-terminus of RsbW fused to eGFP. Using this reporter, the authors show that ATG is more efficiently used as a start codon than ATA (~30% of ATG) and ATT (~0%) explaining the stimulating effect of these mutations on SigB activity. Analysis of start codon usage in the sigB operon genes among 70.000 genomes showed that non-canonical start codons are extremely rare and therefore unlikely to explain virulence differences between different phylogenetic sublineages (which are more common). In a more systematic analysis of start codon usage of 2180 core genome genes, non-canonical start codons were observed for 42 core genome genes, among them plcB (phospholipase C) and lftR (transcriptional regulator), for which the effect of an ATG->GTG exchange was quantified in a reporter assay.

Although the study is interesting and reports on a previously overlooked aspect that may be relevant to the virulence of L. monocytogenes, I consider it to be a preliminary study that needs to be complemented by additional experiments to further demonstrate the relevance of its main findings.

The authors thank reviewer #2 for carefully reviewing the manuscript and raising insightful questions and suggestions. We have revised the manuscript with additional data to support the findings and addressed other concerns raised to improve the data presentation, explanation, and interpretation. To help understanding some responses in relation to the additional data, here are how various MQ140025 strains were re-named (same as revised Table 1):

Name	Source	Sequencing	SigB operon genotype
POI	point of isolation	previsou Illumina sequencing	rsbX N77K, rsbU Q317*
INT	shared to Burgess lab	Nanopore sequencing, this study	rsbX N77K
ANC	derivative of INT		wild type

General concerns:

1. The parental strain used here has lowered SigB-activity due to two rsb mutations. The relevance of this genetic constellation is unknown. How frequent are these mutations among clinical and/or environmental strains?

The authors thank the reviewer for raising this interesting point for discussion. In a few previous studies, the authors have demonstrated high prevalence of loss-of-function mutations in genes encoding positive SigB regulators (*rsbS*, *rsbT*, *rsbU*, *rsbV*, and *sigB*, mentioned at Ln407-411). These mutations occur frequently from *in vitro* evolutionary experiments, food and clinical isolates, and public databases of *L. monocytogenes* genomes. To a large extent these changes explain the variations in stress response profiles and virulence potential of wild *L. monocytogenes* strains. Whether and how these frequencies differ between clinical strains and non-clinical strains is a current research focus in the lab. However the source of isolation in public databases are usually poorly identified, which makes this task challenging. Despite these, the unusually high occurrences of loss-of-function SigB mutations in food/clinical-strains (which constitute the public database) already suggest that they were selected at some stage in these environments by conferring fitness advantage(s). This research direction is now mentioned in the revised manuscript at Ln446-449.

2. How relevant is this phenomenon for SigB+ strains? Does a similar phenomenon (occurrence of non-canonical start codons in rsbW) can be observed in wild type strains during stress exposure to constitutively switch on SigB?

The authors agree with the reviewer that this will be a very interesting experiment to do. The strain MQ140025 was selected for this experiment primarily because it has reduced SigB activity and thus defected host-entry. Therefore, it is highly unlikely that an *in vitro* evolutionary experiment will make this strain more infective than a general Lm strain. The authors have indeed considered evolving a SigB+ strain under a more acidic environment (below pH2.5) however, if successful, this would generate potentially risky gain-of-function mutations in a BSL-2 pathogen. This biosafety consideration is the primary reason we have decided against this experiment. However, through bioinformatic analysis, two other occasions of *rsbW* start codon mutations were observed (Figure 5E) suggesting that

they occur in the environment although the precise selective pressure is unknown. This is elaborated in the revision at Ln311-314.

3. The study is focused on the effect of the rsbW start codon mutations on acid stress survival and expression of few sigB-dependent genes. To make their results more relevant, the authors should demonstrate that non-canonical start codons in rsbW increase virulence.

The authors agree that additional virulence experiments can help to support the clinical relevance of *sigB* operon mutations (*rsbW* in this case). In this study, the effects of elevated SigB activities on acid resistance (represents a challenge typically encountered in human stomach) and *inlA* expression (critical for establishing infection) have been shown, indicating a strong clinical relevance of these mutations. To further demonstrate their effects on virulence, the authors are currently establishing protocols and setting up facilities for macrophage survival assay and epithelial cell invasion assay. These results will be generated and published as part of a subsequent project Ln491-493.

4. To validate their results, the authors need to reconstruct the rsbW start codon mutations in the parental background. Ideally, these mutations also should be transplanted into a SigB+ reference strain to determine their effect on SigB activation in a SigB+ background.

The authors agree that demonstrating the effect of *rsbW* start codon mutation in a SigB+ strain will help to explain the biological relevance of such mutations. The authors have made attempts to swap *rsbW* start codon into reference strains EGD-e (lineage II, CC9) and F2365 (lineage I, CC1) however these attempts failed, presumably due to the growth cost associated with elevated SigB activities as a consequence of these mutations. Instead, the authors generated a MQ140025 strain with wild type *sigB* operon (strain "ANC", wild type *sigB* operon) from the MQ140025 variant used for Nanopore sequencing (strain "INT", *rsbX* N77K). By comparing MQ140025 strains ANC, INT and POI (point of isolation, *rsbX* N77K and *rsbU* Q317*), the authors showed that *rsbW* start codon mutations (ATG-ATT or ATG-ATA) resulted in higher SigB activities, higher acid resistances, and lower growth rates than normal SigB+ strain. They also produce similar effects as loss-of-function mutations in another negative SigB regulator (*rsbX* N77K). These results explained why previous attempts in generating *rsbW* start codon mutants failed: because consecutive growth at 30°C is needed for selecting mutants while these *rsbW* start codon mutants display a growth disadvantage at 30°C. These data demonstrate the biological significance of *rsbW* start codon mutations, and are added as an additional result section (Ln239-270, Figure 4).

5. The section on the occurrence of alternative start codons is interesting but it would gain additional importance if the effect of selected start codon changes in the most interesting genes on virulence would be determined.

The authors agree with reviewer that demonstrating the effects on these alternative start codons on virulence will be very interesting and of further importance. Indeed this is already an on-going investigation in the lab. As mentioned in response to "General concern 3", the authors are setting up facilities and methodologies to test several virulence traits. Meanwhile, this manuscript addresses the significance of *sigB* operon mutations regarding the *in vitro* fitness and their functional relevance to the adaption/evolution trajectory of a clinical *Lm* strain, with interesting discoveries of alternative start codon usage among *Lm* wild isolates. Therefore, the authors intend to publish the full investigation into virulence effect as part of a separate study. This research plan is now mentioned at Ln491-493.

Specific remarks:

Line 177: The authors conclude here that only gadD3 was upregulated in the "LM" group, but at least two out of the LM strains show upregulation of gadT2, too, and gadD3 was also increased in ARD6. This is irritating and needs clarification.

The authors thank reviewer for pointing this out. This statement has been reworded to better describe the observation. It now reads: "Among these three genes only the transcription of SigB-dependent gene *gadD3* in the ARDs was upregulated to similar levels as reference strain F2365." (Ln184-185).

Line 178: The section on ArgR needs rephrasing. It is not clear which changes in the ArgR amount are meant? Changes that could have been deduced from the occurrence of deviations in the aguA1 transcript levels?

The authors agree that this could be confusing, therefore this sentence has been rephrased as: "No significant differences in *aguA1* transcript levels were detected between the ARDs and parental strain, suggesting no changes in ArgR activity had occurred." (Ln185-187)
Fig. 2A-B: More contrast is needed.
The authors have enhanced the contrast of this figure to help distinguish the phenotypical differences (Figure 2).
Line 193: The genome of the MQ strain was re-constructed using long read sequencing data only? Long read data need to be polished by short read data (Illumina) to remove SNPs which are introduced with high frequency in nanopore sequencing data.
The authors agree that this will help to consolidate the findings. The detected differences (presumptive mutations) between genome assemblies from Nanopore sequencing (using strain INT) and previous Illumina sequencing (using strain POI) were already compared by counting the numbers of reads that support the presumptive mutations and that do not support the presumptive mutations. The ambiguous mutations (<99%) were filtered out, leaving a high confidence of the presumptive mutations *rsbU* *317Q (a reversion of the mutant *rsbU* allele to wild-type) and *lmo1979* ΔE128, as we reported in the original submission. To further confirm this, the DNA sample used for Nanopore sequencing was sent for illumina sequencing, and the resulting sequence reads were used for polishing the genome assembly from long read sequencing (Nanopore sequencing). The results were in agreement with the original submission that the INT strain carries *rsbU* *317Q and *lmo1979* ΔE128. The *rsbU* sequence in both strain POI and INT was also separately examined by colony PCR and sanger sequencing, this also confirmed the *rsbU* Q317* in POI and *rsbU* *317Q mutation in INT. The authors have updated the results section accordingly (Ln211-219), while Figure 4 and Table 1 will further help to clarify the sequence of the mutation events.
Line 204: Why did the resequencing of the MQ strain not confirm the rsbX N77K mutation mentioned in the introduction?
The original strain MQ140025 used for previsous Illunima sequencing and evolution experiment (*rsbX* N77K, *rsbU* Q317*) is re-named strain POI, while the MQ140025 strain that was shared to a different laboratory (Burgess lab) for Nanopore sequencing is re-named strain INT. The *rsbX* sequence from strain POI (previous Illumina sequencing) and strain INT (Nanopore sequencing, this study) are consistent, which means the Nanopore seqeuncing confirmed the previously reported *rsbX* N77K genotype. However the previously reported *rsbU* *317 genotype of strain POI was not confirmed in the strain INT (the *rsbU* stop codon at 317 spontaneously reverted back to glutamine codon – wild type). By repeating illumina sequencing for strain INT (taking the same DNA sample used in Nanopore sequencing) and by Sanger sequencing, we confirmed *rsbU* Q317 genotype in strain INT and thus INT only has *rsbX* N77K lesion within *sigB* operon (Figure 4, Table 1, Ln211-219).
Line 216: An explanation is needed that rsbV and rsbW overlap.
The authors have now rephrased this sentence to make this clear, it reads "Since the coding sequences of rsbW and rsbV overlap (17 bp), these mutations produce dual effects: …" (Ln230-231). We have also mentioned this in footnote of Table 1.
Line 239: "was" is missing
This mistake has been corrected, Ln291 .
Table S1: Please include gene names where available. Why is lmo0205 (plcB) or lftR not included in this table? Do they not belong to the conserved core genes? It would be helpful to include data on the frequency of alternative start codons for the 39+2 genes that are explicitly discussed in the text here as well.
The authors thank reviewer for this suggestion. Table S1 has been revised to include the gene names, where available, and the genes with flexible start codon usage. While systematically reorganising the table of conserved genes and their start codon usage, the authors noticed that 85 conserved genes with fixed start codons were accidently omitted during previous column sorting. This correction brought up the total conserved gene numbers to 2265, however it didn't affect the number of genes with flexible start codon usage (39). These corrections have been made in the main text and the Table

S1. Again, the authors appreciate the reviewer for raising this concern, which helped to avoid misleading the readers with incomplete information.
