# Supplementary material for: Translation control by altered start codon usage as a means of modulating the general stress response and virulence in Listeria monocytogenes
Source: PLoS Genet. 2026 Apr 6;22(4):e1011851. doi: 10.1371/journal.pgen.1011851 (PMC13068326; doi:10.1371/journal.pgen.1011851)
Supplement: Attachment — Submitted filename: Point-by-point_R2.pdf [file pgen.1011851.s009.pdf]

Point-by-point response to reviewers:

Additional Editor Comments:

The reviewers are largely satisfied with the revisions and offer just a few minor corrections. Reviewer #2 is still concerned about not being able to introduce the start site mutation into a clean background. However, I am satisfied with the authors' explanation for why this proved impossible and the additional information provided to address this point.

We thank the editor for handling our manuscript and making a decision on this submission. We agree with reviewer 2 that this additional evidence would have further consolidated our findings however with regrate we admit that multiple attempts in constructing such mutation failed. While we will consider this experiment when appropriate genetic tool becomes available in *L. monocytogenes*, we believe the current form of this study provides strong evidence rooting for the conclusion and would be of interests to the readers of *PLOS GENETICS*. Please kindly find point-by-point response to the concerns raised by both reviewers below.

Reviewers' comments:

Reviewer #1: The authors have largely addressed the reviewers' concerns and suggestions in their revised manuscript. I therefore recommend publication of this article.

We thank reviewer 1 for careful evaluation of the revised manuscript and we, again, appreciate all the feedbacks that helped us to improve this study.

Minor edits:

Line 247-248: Figure 1A needs to be changed to Figure 4A.

This mistake has been now corrected.

Line 261: Figure 1A needs to be Figure 4A.

This mistake has been now corrected.

Reviewer #2: The authors have satisfactorily answered most of the points of criticism I raised. However, they leave open the question of whether the generation of rsbW start codon mutations in the POI parental strain using homologous recombination techniques is sufficient to cause the same phenotypic consequences as in the ARD suppressors. Even if technical limitations make such experiments impossible in their case, I consider this proof to

be essential, since mutations can always remain undetected in genome sequencing of suppressor strains, especially duplications, rearrangements, and unstable mutations. Therefore, an important argument needed to support their hypothesis remains missing in my eyes. I leave it to the editor to decide whether an experiment of this kind needs to be included in order to meet the standards of PLOS Genetics.

We thank reviewer 2 for pointing out the importance of demonstrating this observation in a clean genetic background. While the additional data in Figure 4 has supported our conclusion to a considerable extend, it would have been ideal to achieve this in a reference strain. We have made several attempts and failed to introduce such mutation. We will re-consider doing this when better genetic tool becomes available in *L. monocytogenes*.

Reviewer 2 also raised an interesting point regarding the limitations of what can be detected by genome sequencing suppressor mutants. The concern of genome structure variations (e.g. duplications, rearrangements) was part of the reasons we determined the complete genome sequence of strain MQ140025. With the complete genome sequence, the mutational analysis employed in this study (breseq) enables the detection of genome structure variations even from a small fraction of the bacterial population. While no genome structure variation was detected from ARDs in this study, we have succeeded in detecting genome rearrangements and duplications in a separate *L. monocytogenes* project using breseq. However, we agree with reviewer 2 that unstable mutations can be overlooked. Although in this work, we believe the fact that multiple mutants (ARDs) displaying the same phenotype and carrying same/similar mutation would have demonstrated that these *rsbW* disabling mutations are indeed responsible for the elevated SigB activities. Again, we thank reviewer 2 for constructive advice and valuable insights!

Special comments

- Fig. 4D: The legend indicates that the line for the POI strain contains green circles, which, however, cannot be found in the diagram.

In Figure 4D, the survival curve for each strain was denoted as lines with dots at each sampling time point of same color. However for the very acid sensitive POI strain, there was no detectable survival even at 15 min (first sampling time point post treatment) – as shown that it fell below the detection limit (horizontal dotted line). Therefore the only green dot/circle of strain POI is at 0 min (before the acid treatment where survival is 100%), representing the only available data. This data point was brought to front of all other data points that would have otherwise overlapped it. We hope this has addressed this concern but we are happy to further clarify if needed.
